# Stand-alone porous titanium cages for anterior cervical discectomy and fusion: clinical outcomes in a multicenter experience

**DOI:** 10.1007/s10143-026-04316-8

**Published:** 2026-05-04

**Authors:** Flavio Panico, Stefano Colonna, Marco Bozzaro, Andrea Gatto, Luca Ceroni, Ludovico Maria Comite, Salvatore Petrone, Marco Ajello, Nicola Marengo, Emanuele Bavaresco, Nicola Zullo, Diego Garbossa, Fabio Cofano

**Affiliations:** 1 Neurosurgery Unit, Department of Neuroscience “Rita Levi Montalcini” , “Città della Salute e della Scienza” University Hospital, University of Turin, Turin, 10126 Italy; 2 Spine Surgery Unit, Humanitas Gradenigo Hospital, Turin, Italy; 3 Department of Psychology, University of Turin, Turin, 10126 Italy; 4 Spine Surgery Unit, Casa di Cura Città di Bra, Bra, Italy; 5 Department of Neuroscience, University of Turin, Via Cherasco, 15, Turin, 10126 Italy

**Keywords:** Spine surgery, Arthrodesis, Anterior cervical discectomy and fusion, ACDF, Porous titanium, Fusion rate

## Abstract

Background: Porous titanium cages have emerged as a promising alternative to traditional interbody materials in anterior cervical discectomy and fusion (ACDF), potentially enhancing osteointegration while maintaining mechanical stability. This multicenter study evaluates clinical and radiological outcomes following stand-alone ACDF using 3D-printed porous titanium cages. Methods: This retrospective observational study included 120 patients (mean age 54.8 years) who underwent stand-alone ACDF across three tertiary spine centers. Clinical outcomes were assessed using the Numerical Rating Scale (NRS) and Short Form-12 (SF-12). Radiological outcomes at 12 months included fusion, subsidence, migration, and sagittal alignment parameters. Fusion assessment at 12 months was performed using CT or dynamic flexion/extension radiographs. Results: At 12 months, interbody fusion was achieved in 106 patients (88.3%; 95% CI: 81.4%-92.9%). NRS scores significantly improved (mean reduction 4.2 points; p < 0.001), while SF-12 showed no significant change. Cage subsidence occurred in 7 patients (5.8%), with no cases of migration. Cervical sagittal alignment parameters remained stable over time, with no significant differences after correction for multiple comparisons. No significant associations were identified between preoperative variables and adverse radiological outcomes. Conclusions: Stand-alone ACDF with porous titanium cages is associated with high fusion rates, significant pain reduction, and low complication rates at 1 year. These findings support the use of porous titanium cages as a viable option in non-plating cervical fusion strategies, although further comparative studies are warranted to better define their relative performance. Clinical trial number: Not applicable.

## Introduction

Anterior cervical discectomy and fusion (ACDF) is a well-established procedure for treating cervical degenerative conditions, including myelopathy and radiculopathy, in patients unresponsive to conservative therapy [2, 10, 22, 24]. Successful fusion depends on both biological and mechanical factors, among which the choice of interbody material plays a critical role [17, 22].

Autologous iliac crest grafts, historically the gold standard, are limited by donor-site morbidity [8, 12, 23]. Allografts reduce these complications but introduce concerns about immunogenicity, graft variability, and disease transmission [6]. Polyetheretherketone (PEEK) cages have progressively become widely used due to favorable mechanical properties and radiolucency, but their bioinert nature may hinder osseointegration and necessitate supplemental grafting [14, 16, 31].

Standard titanium, carbon fiber or tantalum cages are also widely used [20]. Recently, 3D-printed porous titanium cages have emerged as a promising alternative, offering a trabecular structure that supports bone ingrowth and fusion while preserving mechanical strength and elasticity [13]. Preclinical studies have demonstrated superior bone–implant contact, osteointegration, and fusion kinetics compared to PEEK [3–5, 11, 27]. Early clinical evidence also suggests favorable outcomes, including reduced rates of subsidence, improved maintenance of segmental lordosis, and higher fusion rates [2, 11, 17, 22].

Despite these encouraging preliminary results, current literature on the performance of trabecular titanium cages in ACDF remains limited, particularly for stand-alone constructs without anterior plating or trans-somatic fixation. The primary aim of this multicenter study is to present a clinical experience with stand-alone 3D-printed porous titanium cages, evaluating their performance in terms of clinical outcomes, interbody fusion rates, and maintenance of cervical sagittal alignment.

## Methods

This study was conducted following the Guidelines for Good Clinical Practice and the Declaration of Helsinki (2002) of the World Medical Association. Written informed consent was obtained from all the patients for each diagnostic and surgical procedure.

This multicenter, retrospective, observational descriptive case series, conducted without a control group, analyzed prospectively collected data from adult patients who underwent stand-alone ACDF for degenerative cervical spondylosis from January 2021 to July 2024. Patients with a history of previous cervical fusion surgery, as well as those for whom complete clinical history and postoperative follow-up data were not available, were excluded from the study. All surgical procedures were performed by experienced spine neurosurgeons from the Molinette - CTO University Hospital – Turin (Italy), Casa di Cura Città di Bra – Bra (Italy), and Humanitas Gradenigo Hospital – Turin (Italy).

### Study design

All patients included in the study underwent a standardized preoperative assessment to identify relevant risk factors, including smoking history, obesity (defined as BMI > 30), diabetes mellitus, osteoporosis or osteopenia confirmed by endocrinological evaluation, and inflammatory bone disease diagnosed by a rheumatologist. Perioperative data collection included the number of surgically treated levels, and the length of the postoperative hospital stay. All patients received trabecular titanium interbody cages (Oyster^®^ ACIF, Silony Spine, Frauenfeld, Switzerland) without anterior plating or trans-somatic anchoring screws. No graft material was placed inside the interbody cages, as their design does not allow adequate space for graft accommodation.

Postoperative follow-up was conducted at 1 month and 1 year after surgery. At each follow-up visit, clinical evaluation was performed and supplemented with standing lateral cervical radiographs to assess radiological outcomes.

### Postoperative outcomes

Clinical outcomes were assessed by comparing pre- and postoperative scores at each follow-up using the 12-Item Short Form Health Survey (SF-12) and the Numerical Rating Scale (NRS), evaluating functional status and pain, respectively. Postoperative complications were documented, including surgical site infection, dysphagia, postoperative hematoma, pseudarthrosis, and the need for revision surgery.

Radiological outcomes were evaluated based on evidence of interbody fusion at the 1-year follow-up. Fusion was assessed using radiographic criteria consistent with Bridwell grades I-II on CT evaluation, with fused cases defined by the presence of trabecular bone bridging at the treated level located anteriorly and/or posteriorly to the implanted cage, and absence of radiolucency around the implant. In patients evaluated with dynamic flexion/extension radiographs, fusion was defined as the absence of motion at the treated level.

Secondary radiological endpoints included the presence of cage subsidence and implant migration. Subsidence was defined as implant penetration of > 2 mm into the adjacent vertebral endplate beyond the cortical margin. Additionally, cervical sagittal alignment parameters were assessed and compared at 1-month and 1-year follow-ups, including the segmental angle at the treated level, global C2-C7 cervical lordosis, T1 slope, and C2-C7 sagittal vertical axis (SVA). All radiological measurements were independently performed by two observers. In cases of agreement, values were accepted directly; in cases of discrepancy, a consensus reading was performed to reach a shared measurement.

### Statistical analysis

Descriptive statistics were reported as mean ± standard deviation for continuous variables and as frequencies with percentages for categorical variables. The Kolmogorov-Smirnov test was used to assess the normality of distribution for continuous variables. Paired samples t-tests were applied to compare parametric continuous variables measured at two different time points within the same subjects. Associations between categorical variables were analyzed using the Chi-square test. Cramér’s V was calculated to quantify the strength of association, with values interpreted as follows: 0.01–0.30 = weak, 0.31–0.60 = moderate, 0.61–0.90 = strong, and > 0.90 = very strong association.

To provide a measure of estimate precision, 95% confidence intervals (95% CI) were calculated for the primary radiological outcome (fusion rate). Standardized effect sizes were computed for pre-post comparisons of clinical outcomes using Cohen’s d. Correction for multiple comparisons across radiological parameters was performed using the Bonferroni method. Additionally, exploratory univariate logistic regression analyses were performed to assess potential associations between preoperative variables and adverse radiological outcomes, reporting odds ratios (OR) with 95% confidence intervals.

A p-value ≤ 0.05 was considered statistically significant. All analyses were performed using IBM SPSS Statistics for Windows, Version 26.0 (IBM Corp., Armonk, NY, USA).

## Results

### Preoperative data

A total of 120 patients were included in the study, with a mean age at the time of surgery of 54 years (Table 1). Among them, 38 patients (31.6%) were treated for cervical disc herniation, 24 (20.0%) for foraminal and/or central canal stenosis, and 58 (48.4%) for degenerative cervical myelopathy.

**Table 1:** Complete demographic and preoperative data

Number of patients	120
Age, mean ± SD	54.8 ± 12.1
Gender, n (%)	
Male	56 (46.7%)
Female	64 (53.3%)
Preoperative diagnosis, n (%)	
Cervical disc herniation	38 (31.6%)
Foraminal and/or canal stenosis	24 (20.0%)
Degenerative myelopathy	58 (48.4%)
Preoperative comorbidities, n (%)	
Smoking	29 (24.1%)
BMI > 30	17 (14.1%)
Diabetes	10 (8.3%)
Inflammatory osteopathy	7 (5.8%)
Osteoporosis/osteopenia	6 (5.0%)
Treatment levels, n (%)	
1 level	82 (68.3%)
2 levels	38 (31.7%)
Length of hospitalization, mean days	2.6 ± 0.9

Regarding preoperative risk factors, 29 patients (24.1%) had a history of smoking, 17 (14.1%) were classified as obese (BMI > 30), and 10 (8.3%) had diabetes mellitus. Inflammatory osteopathy and osteoporosis were reported in 7 (5.8%) and 6 (5.0%) patients, respectively. The mean postoperative hospital stay was 2.6 ± 0.9 days.

### Clinical outcome

Compared to preoperative values, clinical follow-up showed a significant reduction in NRS scores (7.4 ± 2.8 vs. 3.3 ± 2.8), whereas SF-12 scores remained substantially unchanged (31.7 ± 3.1 vs. 31.2 ± 2.5). The mean difference for SF-12 was 0.46 (95% CI: -0.30 to 1.24; Cohen’s d = 0.152; *p* = 0.169), while NRS scores showed a significant reduction with a mean difference of 4.20 (95% CI: 3.35–5.05; *p* < 0.001), corresponding to a large effect size (Cohen’s d = 1.23).

Transient postoperative dysphagia occurred in 6 patients (5.0%) and resolved with steroid therapy. No cases of pseudoarthrosis requiring revision surgery, surgical site infection, or adjacent segment disease were documented. One patient who underwent C4-C5 ACDF for degenerative myelopathy required subsequent posterior laminoplasty due to suboptimal spinal cord decompression and persistent symptoms (Table 2).

**Table 2:** Complete data on clinical assessment and postoperative complications

SF-12 score, mean ± SD (median)	
Preoperative	31.7 ± 3.1 (31)
Postoperative, last follow-up	31.2 ± 2.5 (31)
NRS score, mean ± SD (median)	
Preoperative	7.4 ± 2.8 (8)
Postoperative, last follow-up	3.3 ± 2.8 (3)
Postoperative complication, n (%)	
Transient dysphagia	6 (5.0%)
Postoperative hematoma	1 (0.8%)
Revision surgery	1 (0.8%)
Infection	0 (0%)
Adjacent segment syndrome	0 (0%)
Pseudoarthrosis	0 (0%)
Total	8 (6.6%)

### Radiological outcome

**Fig. 1:**
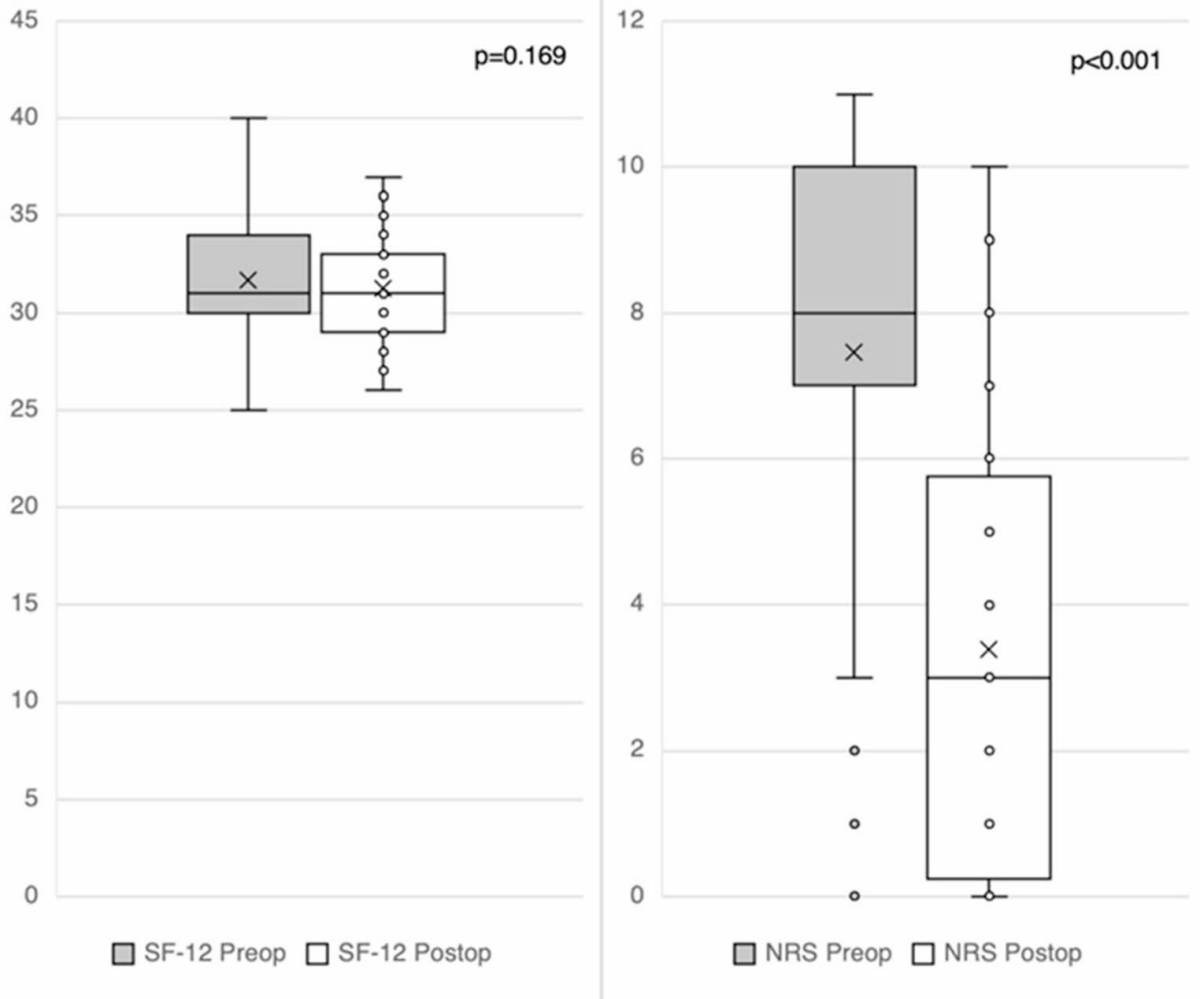
Boxplots comparing preoperative and postoperative clinical outcomes in terms of SF-12 (*p* = 0.169) and NRS (*p* < 0.001) scores

Postoperative radiographs demonstrated successful interbody fusion in 106 patients (88.3%; 95% CI: 81.4%-92.9%). Fusion assessment at 12 months was performed by CT scan in 33 patients (27.5%), while the remaining patients were evaluated using dynamic flexion/extension radiographs. Cage subsidence was observed in 7 patients (5.8%), with no cases of cage migration (Table 3; Fig. 1).

**Table 3:** Complete data on primary and secondary radiological outcomes

Segmental Lordosis, ° (median)	
1-month postoperative	10.4° ± 3.7° (10.5°)
12-months postoperative	10.2° ± 5.0° (9.4°)
C2-C7 Lordosis, ° (median)	
1-month postoperative	15.1° ± 9.3° (13.75°)
12-months postoperative	14.7° ± 8.4° (12.5°)
T1 Slope, ° (median)	
1-month postoperative	26.5° ± 7.6° (25°)
12-months postoperative	26.4° ± 8.3° (25°)
C2-C7 SVA, mm (median)	
1-month postoperative	25.6 ± 15.4 (25.5)
12-months postoperative	25.5 ± 14.8 (22.5)
Intersomatic fusion, n (%)	106 (88.3%)
Cage subsidence, n (%)	7 (5.8%)
Cage mobilization, n (%)	0 (0%)

**Fig. 2:**
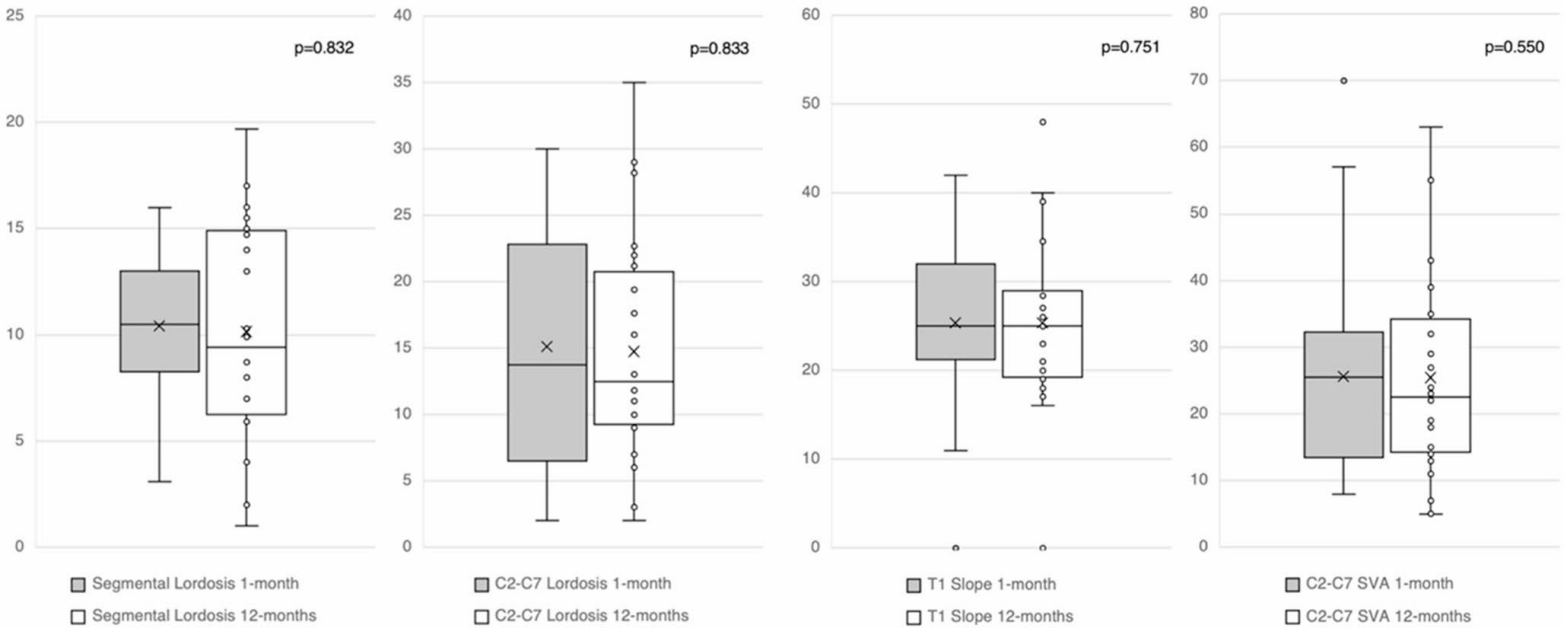
Boxplots comparing preoperative and postoperative radiological sagittal balance outcomes (*p* > 0.05)

No significant differences were found between the 1-month and 12-month postoperative measurements for segmental angle (10.4° vs. 10.2°; mean difference 0.2; 95% CI: -1.33 to 1.88; *p* = 0.832), C2-C7 lordosis (15.1° vs. 14.7°; mean difference 0.4; 95% CI: -1.74 to 2.47; *p* = 0.833), T1 slope (26.5° vs. 26.4°; mean difference 0.1; 95% CI: -1.69 to 1.76; *p* = 0.751), and C2-C7 sagittal vertical axis (25.6 mm vs. 25.5 mm; mean difference 0.1; 95% CI: -3.13 to 3.38; *p* = 0.550). These findings remained non-significant after correction for multiple comparisons using the Bonferroni method (Table 4; Fig. 2).

**Table 4:** Pre- and postoperative clinical and radiological outcomes with mean differences, 95% CI, and effect sizes

Clinical outcome	Preoperative	Postoperative (12-months)	Mean difference (95% CI)	Cohen’s d	*p*-value
SF-12 score	31.70	31.23	0.46 (-0.30 – 1.24)	0.15	0.169
NRS score	7.45	3.25	4.20 (3.35 – 5.05)	1.23	<0.001
Radiological outcome	Postoperative (1-month)	Postoperative (12-month)	Mean difference (95% CI)		*p*-value
Segmental Lordosis, °	10.4°	10.2°	0.2 (-1.33 – 1.88)		0.832
C2-C7 Lordosis, °	15.1°	14.7°	0.4 (-1.74 – 2.47)		0.833
T1 Slope, °	26.5°	26.4°	0.1 (-1.69 – 1.76)		0.751
C2-C7 SVA, mm	25.6	25.5	0.1 (-3.13 – 3.38)		0.550

Univariate logistic regression analyses did not identify any significant associations between preoperative variables (including number of treated levels, BMI, smoking status, osteoporosis, inflammatory osteopathy, diabetes, and sex) and adverse radiological outcomes (Table 5).

**Table 5:** Association between preoperative variables and postoperative radiological outcomes using Cramer’s V and univariate logistic regression (LR)

Preoperative variable	Cramér’s V	*p*-value(Chi-square)	OR (95% CI)	*p*-value(LR)
Number of treatment levels	0.019	0.915	0.88 (0.08 – 9.28)	0.915
BMI > 30	0.312	0.119	NE	0.994
Smoking	0.238	0.243	3.25 (0.42 – 24.84)	0.256
Osteoporosis	0.083	0.632	NE	0.995
Inflammatory osteopathy	0.083	0.632	NE	0.995
Diabetes	0.020	0.909	1.15 (0.10 – 12.61)	0.909
Sex	0.072	0.678	0.68 (0.11 – 4.05)	0.679

*NE* not estimable

**Fig. 3:**
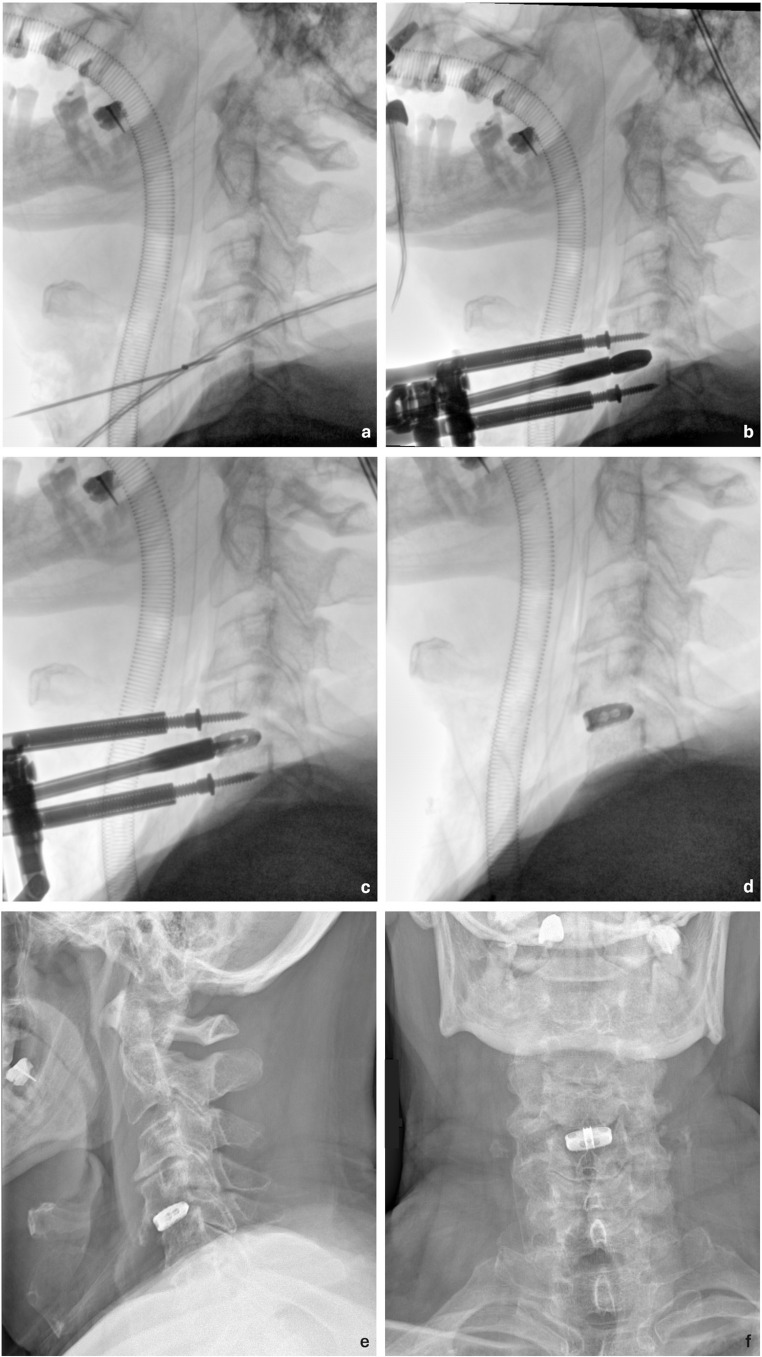
Illustrative case of anterior cervical discectomy and fusion using a stand-alone porous titanium cage with intraoperative and postoperative imaging. (**a**) Level localization using an intradiscal probe. (**b**) Pin placement and discectomy, a trial spacer was inserted into the intervertebral space to determine the appropriate cage size. (**c**) Definitive cage implantation using the cage holder. (**d**) Final intraoperative image after completion of the procedure and pin removal. (**e**, **f**) Postoperative radiographs in lateral and anteroposterior projections, respectively

An illustrative case of ACDF with intraoperative and postoperative radiographic images is shown in Fig. 3.

## Discussion

This retrospective multicenter descriptive case series evaluated the clinical and radiographic outcomes of porous titanium interbody cages used as a stand-alone device for ACDF in patients with degenerative cervical spine disease. The results suggest encouraging clinical outcomes, with a reduction in pain and a low complication rate. While these findings are promising, they should be interpreted with caution given the descriptive, non-comparative nature of this study.

Porous titanium cages have been suggested to provide potential advantages over PEEK and structural bone grafts, including superior osteointegration, an elastic modulus closer to native bone, and avoidance of allograft-related risks [3, 11, 13, 27]. In vitro and preclinical studies consistently show enhanced osteoinductive and osteoconductive properties of titanium trabecular surfaces compared with PEEK and other metals, with increased cell activity and early bone formation [4, 5, 7, 16, 19]. Histological analyses of explanted cervical cages demonstrate complete osteointegration with mature lamellar bone at the interface [5], while 18 F-NaF PET/CT studies confirm robust osteogenic activity after lumbar arthrodesis, validating the biological efficacy of porous titanium in vivo [9, 26].

In our cohort, cage subsidence occurred in 5.8% of cases, with no implant migration, suggesting acceptable mechanical stability even without anterior plating or self-locking screws. These findings appear consistent with those reported in recent literature on porous titanium cages in ACDF. Singh et al. reported reduced subsidence and better maintenance of segmental lordosis compared with allografts, alongside a fusion rate of 83% and similar clinical outcomes [22], while Arts et al. demonstrated a fusion rate of 91% with clinical improvement at one year in a prospective study of porous cervical cages [2]. In the present series, we observed a high fusion rate (88.3%) and low complication profile, which appear consistent with these previously reported findings. However, the present study is a single-arm, non-comparative analysis, and therefore no direct conclusions can be drawn regarding the superiority of porous titanium cages over other interbody materials. It should also be acknowledged that fusion assessment was based mainly on plain radiographs, which are known to underestimate true fusion rates compared with CT evaluation, representing a methodological limitation that may partially account for the observed rate.

Implant design plays a key role, as both experimental and clinical studies show that geometry and internal architecture influence load distribution and subsidence risk [18, 28]. Cages with porous gradient structures or tapered profiles reduce endplate stress and improve stability [18, 21]. In addition, in the lumbar spine, multiple studies have reported favorable radiological outcomes with porous titanium compared with PEEK; however, these findings derive from different clinical settings and should not be interpreted as direct confirmation of our results [1, 15, 21, 30].

NRS scores improved after surgery (mean reduction 4.2 points, *p* < 0.001), suggesting a clinically relevant reduction in pain. SF-12 scores showed no significant change, likely due to the short follow-up or a “ceiling” effect in otherwise healthy patients, though a trend toward functional recovery was observed. No pseudoarthrosis or infections occurred; one patient required reoperation for insufficient decompression, unrelated to the implant.

Cervical sagittal alignment remained stable over time, confirming that stand-alone porous titanium cages preserve spinal balance in the medium term. These findings are consistent with reports from lumbar series, in which porous cages likewise maintained stability and sagittal alignment [25]. Traditional risk factors such as smoking, obesity, diabetes, and osteoporosis showed no significant association with adverse clinical or radiographic outcomes. This finding suggests that satisfactory fusion may be achieved with porous cages even in the presence of traditionally unfavorable conditions [29].

### Limitations

This study has some limitations. Its retrospective design introduces potential selection bias and limits causal inference. Fusion assessment was primarily based on dynamic radiographs, while CT was available only in a subset of patients; this may have led to an underestimation of the true fusion rate. The absence of a control group treated with alternative interbody materials prevents any direct comparison and limits the generalizability of the findings. Comparative studies, including randomized controlled trials or well-matched cohort analyses, are needed to more clearly define the relative performance of different implants. Finally, the exploratory logistic regression analyses should be interpreted with caution given the limited number of adverse events, which may have resulted in unstable estimates. The 12-month follow-up captures only medium-term outcomes and does not provide information on long-term events such as adjacent segment degeneration or construct longevity.

## Conclusion

In this descriptive case series, porous titanium cervical implants for ACDF showed promising clinical outcomes after surgery, with apparently favorable results in terms of subsidence and fusion rates, and a negligible risk of displacement despite the absence of plates or self-locking screws for cage fixation. These findings suggest a potential role for porous implants in minimally invasive, non-plating surgical strategies, though they should be interpreted with caution given the descriptive, non-comparative nature of this study. Comparative studies and prospective trials with long-term follow-up are warranted to confirm these preliminary results and more precisely define the role of these devices compared with traditional interbody materials. 

## Data Availability

The data supporting the findings of this study are available from the corresponding author upon reasonable request.
